# Adherence to reduced-polluting biomass fuel stoves improves respiratory and sleep symptoms in children

**DOI:** 10.1186/1471-2431-14-12

**Published:** 2014-01-17

**Authors:** Roberto A Accinelli, Oscar Llanos, Lidia M López, María I Pino, Yeny A Bravo, Verónica Salinas, María Lazo, Julio R Noda, Marita Sánchez-Sierra, Lacey Zárate, Joao da Silva, Fabiola Gianella, Leila Kheirandish-Gozal, David Gozal

**Affiliations:** 1 Laboratorio de Respiración del Instituto de Investigaciones de la Altura, Universidad Peruana Cayetano Heredia, Lima, Perú; 2 Hospital Nacional Cayetano Heredia, Lima, Perú; 3 Facultad de Medicina Alberto Hurtado, Universidad Peruana Cayetano Heredia, Lima, Perú; 4 Section of Pediatric Sleep Medicine, Department of Pediatrics, and Comer Children’s Hospital, Pritzker School of Medicine, The University of Chicago, Chicago, IL, USA; 5 Department of Pediatrics, University of Chicago, 5721 S. Maryland Avenue, MC 8000, Suite K-160, Chicago, IL 60637, USA

**Keywords:** Biomass fuel pollution, Sleep, Respiration, Kitchen stoves

## Abstract

**Background:**

Symptoms of sleep apnea are markedly increased in children exposed to smoke from biomass fuels and are reduced by kitchen stoves that improve indoor biomass pollution. However, the impact of adherence to the use of improved stoves has not been critically examined.

**Methods:**

Sleep-related symptom questionnaires were obtained from children <15 years of age in 56 families residing in the communities of Lliupapuquio, Andahuaylas province in Peru before and 2 years after installation of less-polluting Inkawasi cooking stoves.

**Results:**

82 children with lifetime exposures to indoor fuel pollution were included. When compared to those alternating between both types of stoves or those using traditional stoves only, those children who exclusively used Inkawasi cooking stoves showed significant improvements in sleep and respiratory related symptoms, but some minor albeit significant improvements occurred when both stoves were concomitantly used.

**Conclusions:**

Improvements in respiratory and sleep-related symptoms associated with elevated indoor biomass pollution occur only following implementation and exclusive utilization of improved kitchen stoves.

## Background

Indoor biomass pollution is one of the most important sources of pollution particularly in the developing world. [1] Indoor fuel sources can be either solid, liquid, or gas combustibles, with biomass fuel and coal constituting by far the most frequently used solid fuels. Biomass fuel refers to plant or animal-derived material products (wood, agriculture waste, and dung) burned for cooking or heating purposes. [2,3] In 2007, solid fuel was used in 42% of all households worldwide, and in 76% of rural households. [4] In developing countries, women and young children are the most exposed to this form of air pollution because they spend the greatest proportion of time near the domestic hearth, while this is further exacerbated in highland areas, since people spend more time indoors because of the cold climate [5-8].

In-house exposures to biomass pollution have been associated with an increased prevalence of lung diseases, including COPD [9-17], recurrent acute respiratory tract infections in children [18-20], lung cancer [21], asthma [22,23], tuberculosis [24-26], interstitial lung disease [27,28] and non pulmonary disease, such as cataracts, nasopharyngeal cancer, ischemic heart disease and cor pulmonale [29]. However, the potential associations between biomass indoor pollution and sleep-disordered-breathing (SDB) have not been as extensively explored. Indeed, in a recent study from our group, we showed a significant increase in the prevalence of symptoms of SDB in children exposed to indoor biomass fuel pollution [30]. However, the impact of adherence to implementation and installation of higher efficiency home based stoves that reduce indoor pollution was not examined. In this context, we took advantage of an initiative aimed at replacing conventional home based stoves with modified kitchen stoves that reduce biomass indoor pollution by more than 95% to assess the impact of such intervention on sleep and SDB symptoms, while monitoring adherence to such intervention.

## Methods

The study was approved by the Human Subject Ethics Committee of the University Cayetano Heredia (registration code #56758). Informed consent was obtained from the parents, and age appropriate assent was also obtained from the children. We performed a prospective survey of residents in Lliupapuquio, Andahuaylas-Peru, a small village located at 3557 meters above sea level, who were beneficiaries of Program Juntos, a Peruvian National initiative against poverty. We included children less than 15 years of age, who had a lifetime exposure to traditional high biomass pollution generating home kitchen stoves. A total of 56 families were identified during a pilot phase intervention to install the Inkawasi stove, a Peruvian designed stove with an external exhaust that has conclusively been demonstrated to reduce particulate matter concentrations (PM 2.5) by 74% when compared to traditional stoves, while using 50% less wood combustible material, as well as displaying a superior safety profile during daily use [31].

Two years after the installation and after confirming the adequate operation of the new stoves, we performed a follow-up survey. In the village, a proportion of the families who received the Inkawasi stoves never used them and preferred to continue using their traditional stoves. A second group opted for the concomitant use of either the traditional stoves or the new stoves, and a third group of families exclusively used the new Inkawasi stoves.

Both pre- and post-intervention surveys were conducted through a structured interview of the parents, using a previously validated questionnaire [32-34]. This questionnaire is particularly focused on respiratory and sleep-associated disorders and their symptoms. However, we did not implement any specific preferential weighting of questionnaire items as previously reported in a US-based population [34]. The major questions for this study included whether the child snored or not, and if so, the severity of snoring (loudness and frequency). All sleep-related questions used Likert-type responses “never” [0], “rarely” (once per week)[1], “occasionally” (twice per week) [2], “frequently” (three to four times per week) [3], and “almost always” (>4 times per week) [4] for the preceding 3-month timeframe. The overall scores to problems during sleep were designated from each of the answers to questions pertaining to sleep and divided by the number of questions. Other answer categories of Likert-type order were for bedtime: “7:00–8:00 p.m.,” “8:00–9:00 p.m.,” “9:00– 10 p.m.,” “10:00–11:00 p.m.,” and “after 10 p.m.”; for wake up time: “5:00–6:00 a.m.,” “6:00–6:30 a.m.,” “6:30–7 a.m.,” “7:00–7:30 a.m.,” and “after 7:30 a.m.”; for loudness of snoring: “mildly quiet” [0], “medium loud” [1], “loud” [2], “very loud” [3], “extremely loud” [4]; for child’s room: “sleep alone,” “share with 1,” “share with 2,” “share with 3,” and “share with >3”; and for all other items binary answer categories were applied. Likert values were treated as continuous variables for comparison purposes, and response scores addressing related symptoms were collapsed and treated as a single score.

Questions on adherence regarding the use of new improved stoves were developed and implemented only during the follow-up visit.

Data were analyzed using SPSS statistical software (version 17.0, Chicago, IL). McNemar tests or Chi-square test with Fisher Exact correction were used to perform paired comparisons of qualitative variables before and after kitchen stove change. A p-value of less than 0.05 was considered to be statistically significant.

## Results

Eighty-two children with lifetime exposures to biomass fuel indoor pollution were included. The mean age was 8.3 ±3.2 years, ranging from 2 to 14 years, and the cohort included 40 boys (48.8%). Of the 82 children, 38 were in households in which only the new Inkawasi stove stoves were available, 19 had access to both the Inkawasi stoves and traditional stoves operating concomitantly in their houses, and 25 children continued to exclusively use the traditional stoves despite having the new Inkawasi stoves installed.

The prevalence of respiratory symptoms for the whole cohort was very high during the initial visit as follows: nasal congestion (40%), frequent colds (41.3%), hyperactivity (26.9%), frequent repetitive movements during sleep (35.4%), sore throat (38%), night time awakenings (42.3%), daytime sleepiness (21.8%), and falling asleep at school (11.7%). In follow-up survey, when the 25 children who did not use the new Inkawasi kitchen stoves were omitted from the analyses (Table 1), the remaining 57 children demonstrated statistically significant reductions in the frequency of sore throat (44.4% vs. 25.9%; p < 0.05), headache at awakening (43.4% vs. 22.6%; p < 0.05), and nightmares (48.1% vs. 25.9%; p < 0.05). A statistically significant improvement in easiness to fall asleep was also found (29.6% vs. 55.6% p < 0.01). When the 19 children who were not exclusively using the modified stove were further excluded from the intervention group, the remaining 38 children continued to demonstrate improvements in ease to fall asleep (19.4% vs. 50%; p < 0.02), and in the frequency of sore throat symptoms (47.2% vs. 22.2%; p < 0.05), as well as improvements in the willingness to go to sleep (51.4% vs. 77.1%; p < 0.05) (Table 1). In fact, the children who exclusively used the improved Inkawasi kitchen stoves exhibited significant improvements in the largest number of questionnaires items (n = 6/28; Table 1) compared to those alternating use of the 2 stoves (n = 1/28; p < 0.05), as well as those not using the improved stoves (0/28; p < 0.02).

**Table 1:** Effects of frequency of use of improved Inkawasi cooking stoves on the itemized symptoms included in the questionnaire before and 2 years after stove installation

**Symptom**	**Improved Inkawasi stove use only**					**Traditional stove use only**					**Mixed Inkawasi and traditional stove use**				
	**(n = 38)**					**(n = 25)**					**(n = 19)**				
	**Before**		**After**		**p value**	**Before**		**After**		**p value**	**Before**		**After**		**p value**
	**n**	**%**	**n**	**%**		**n**	**%**	**n**	**%**		**n**	**%**	**n**	**%**	
**Reduced appetite**	16	47.1	15	44.1		17	70.8	15	62.5		6	35.3	6	35.3	
**Ear infections**	4	12.1	5	15.2		2	9.5	4	19		3	16.7	0	0	
**Frequent colds**	18	54.5	11	33.3		11	45.8	7	29.2		2	11.1	4	22.2	
**Nasal congestion**	17	50	13	38.2		7	29.2	9	37.5		6	35.3	1	5.9	
**Attention deficits**	16	48.5	11	33.3		13	54.2	5	20.8		9	50	5	27.8	
**Hyperactivity**	10	28.6	11	31.4		7	28	9	36		4	22.2	8	44.4	
**Snoring**	4	11.4	4	11.4		7	28	6	24		2	11.1	4	22.2	
**Repetitive movements during sleep**	11	30.6	18	50		7	28	9	36		10	55.6	8	44.4	
**Problems during sleep**	20	53.9	1	2.6	<0.0005	5	20.8	5	20.8		10	52.6	1	5.3	<0.005
**Respiratory effort during sleep**	3	8.8	2	5.9		0	0	3	12.5		0	0	1	5.6	
**Stops breathing during sleep**	4	11.8	2	5.9		1	4.2	2	8.3		1	5.6	2	11.1	
**Needs being shaken to breathe during sleep**	2	6.1	4	12.1		2	8.7	3	13		1	5.9	0	0	
**Ease falling asleep**	7	19.4	19	50	<0.02	9	37.5	14	58.3		9	50	12	66.7	
**Sore throat**	17	47.2	8	22.2	<0.05	6	24	7	28		7	38.9	6	33.3	
**Enuresis**	4	11.1	5	13.9		6	24	4	16		4	22.2	2	11.1	
**Wakes up to urinate**	13	35.1	15	40.5		9	36	13	52		10	55.6	8	44.4	
**Easiness to wake up**	21	56.8	30	80.7	<0.05	13	54.2	21	87.5	<0.04	9	50	10	55.6	
**Willingness to go to bed**	18	51.4	27	77.1	<0.05	21	84	20	80		12	66.7	11	61.1	
**Not rested after sleep**	11	30.6	15	41.7		4	16	6	24		2	11.1	4	22.2	
**Wakes up during the night**	15	42.9	7	20		9	36	10	40		9	50	10	55.6	
**Speaks during sleep**	12	34.3	10	28.6		11	44	10	40		4	22.2	5	27.8	
**Sleepwalking**	1	2.8	1.	2.8		2	8	1	4		2	11.1	0	0	
**Sits while asleep**	5	14.3	3	8.6		5	20.8	3	12.5		2	11.1	3	16.7	
**Nightmares**	17	47.2	10	27.8		12	48	8	32		9	50	4	22.2	
**Morning headache**	15	41.7	6	19.8	<0.02	7	28	7	28		8	47.1	6	32.6	
**Mouth breathing**	5	13.9	5	13.9		2	8	3	12		2	11.1	0	0	
**Daytime sleepiness**	7	20	8	22.9		6	24	6	24		4	22.2	0	0	
**Sleepiness at school**	4	11.8	3	8.8		4	16	3	12		1	5.6	0	0	
**Worried about sleep problems**	3	8.8	4	11.8		4	18.2	0	0		0	0	2	11.1	

## Discussion

In the present study, we confirm previous findings that children who have endured lifelong exposures to biomass fuel indoor pollution display an inordinately high prevalence of sleep and respiratory symptoms, including SDB symptoms [30]. Furthermore, we now show that the magnitude of improvement in a selected number of these symptoms, particularly sleep-related symptoms, emerges only after changing the stoves to those with improved, less polluting characteristics. Interestingly, even partial use of the new and improved Inkawasi stove was associated with some degree of improvements, albeit not as prominent or extensive as those occurring when the new improved stoves were exclusively used.

In the context of the current study, we found a remarkably similar prevalence of respiratory and sleep symptoms when compared to our previous study with the exception of snoring (16.7% vs. 52.5%) [30]. However, the magnitude of the reduction of those symptoms in the present study was not as prominent, even though the same survey tool was used, and children in both studies had similar ages and ethnicity. Therefore, it is possible that the differences in response may reflect the mixed utilization patterns of the new stoves in the present study compared to the comprehensive implementation of the Inkawasi stove in our previous intervention [30].

In a parallel study of this population, the degree of indoor pollution was markedly reduced when consistent, correct, and exclusive use of the improved Inkawasi kitchen stoves was implemented. Compared to traditional stoves, the particulate matter concentration levels decreased by 74% and the CO concentration level decreased by 97%. Of the new stoves installed here, we found that one year after installation, 78% had deteriorated grills, and 61% had deteriorated combustion chambers, both of these factors clearly reducing the efficiency of the new stoves. As such, the differences in the responses recorded in this study and the preceding study probably reflect some of these technical and equipment-related issues. Appropriate utilization and maintenance of the higher efficiency stoves are critically important factors for the correct operation of the stoves, and for the anticipated emergence of improvements in respiratory symptoms. In a study performed in African children who intermittently cooked inside their residences or outdoors, cooking with solid fuels in the absence of a chimney or hood was associated with a statistically significant adjusted hazard ratio of 2.68 (95% CI: 1.38 to 5.23) for the emergence of symptoms of respiratory infection. Although outdoor cooking is less harmful than indoor cooking, overall, stove ventilation efficiency emerges as the most significant determinant of acute lower respiratory infection mortality [35]. Similar studies have been reported in other countries, such as in India [36], Nepal [37] and Pakistan [38]. In a 2011 meta-analysis of 25 studies, the overall pooled odds ratios indicate significant associations between indoor biomass fuel pollution and acute respiratory infection in children (OR 3.53, 95% CI 1.94 to 6.43) [23]. In our study, a most salient finding was that compliance in the use of the improved stoves was a major determinant of the outcomes associated with the stove replacement intervention. In addition to the correct and exclusive use of the Inkawasi stoves, appropriate maintenance of such stoves over time appears to be important in securing improved outcomes.

Some limitations of the present study deserve mention. First, the study did not implement any objective measures of sleep or lung function, and such approach would clearly be needed in future studies. Secondly, quantitative monitoring of environmental pollution in each of the households throughout the duration of the study would be desirable and is planned in the context of subsequent initiatives aiming to further spread the implementation of reduced biomass in-house pollution in poor villages. Finally, the study was not powered to identify differences in most of the questionnaire items, since the frequency of positive responses for several of the questionnaire items in the pre-implementation group was low, and therefore given the small number of children in all 3 groups, demonstration of a statistically significant decrease would be precluded under such circumstances.

## Conclusions

In summary, the improvements in sleep-related symptoms as previously observed following adequate and supervised implementation of modified kitchen-stoves were not as significant without the correct and exclusive use of such less-polluting stoves. Reduced adherence to the use of improved stoves for biomass fuels and inadequate operation and maintenance of the stoves emerged as determining factors associated with reduced effectiveness of the intervention. There is no doubt that changing from traditional biomass fuel stoves to improved less polluting stoves is of main importance in reducing indoor air pollution associated symptoms. However, such interventions need to be coupled with implementation of measures that will ensure permanent and exclusive use of the new stoves along with appropriate maintenance training. Otherwise, such well-intentioned programs aiming to reduce sleep and respiratory morbidity due to biomass fuel indoor pollution may either fail or underperform.

## Competing interests

The authors declare that they have no competing interests.

## Authors’ contributions

All of the authors have made substantial contributions to the study. RAA: conception and design of the study, interpretation of data, and drafting of the article. OL: analysis and interpretation of data, and drafting the article. LL: design of the study, acquisition of data, and drafting of the article. YB, VS, ML, JN, MS-S, LZ, JS and FG: design of the study and acquisition of data. LKG and DG performed critical evaluation and interpretation of the data, and revised the manuscript critically for important intellectual content. All authors have reviewed and approved the final version of the manuscript.

## Pre-publication history

The pre-publication history for this paper can be accessed here:

http://www.biomedcentral.com/1471-2431/14/12/prepub
